# Artificial intelligence in clinical decision support and the prediction of adverse events

**DOI:** 10.3389/fdgth.2025.1403047

**Published:** 2025-05-30

**Authors:** S. P. Oei, T. H. G. F. Bakkes, M. Mischi, R. A. Bouwman, R. J. G. van Sloun, S. Turco

**Affiliations:** ^1^ Biomedical Diagnostics Lab, Department of Electrical Engineering, Eindhoven University of Technology, Eindhoven, Netherlands; ^2^ Anesthesiology, Catharina Hospital, Eindhoven, Netherlands

**Keywords:** artificial intelligence, clinical decision support, interpretable AI, trustworthy AI, clinical translation, deskilling

## Abstract

This review focuses on integrating artificial intelligence (AI) into healthcare, particularly for predicting adverse events, which holds potential in clinical decision support (CDS) but also presents significant challenges. Biases in data acquisition, such as population shifts and data scarcity, threaten the generalizability of AI-based CDS algorithms across different healthcare centers. Techniques like resampling and data augmentation are crucial for addressing biases, along with external validation to mitigate population bias. Moreover, biases can emerge during AI training, leading to underfitting or overfitting, necessitating regularization techniques for balancing model complexity and generalizability. The lack of interpretability in AI models poses trust and transparency issues, advocating for transparent algorithms and requiring rigorous testing on specific hospital populations before implementation. Additionally, emphasizing human judgment alongside AI integration is essential to mitigate the risks of deskilling healthcare practitioners. Ongoing evaluation processes and adjustments to regulatory frameworks are crucial for ensuring the ethical, safe, and effective use of AI in CDS, highlighting the need for meticulous attention to data quality, preprocessing, model training, interpretability, and ethical considerations.

## 1 Introduction

The increasing use of artificial intelligence (AI) has proven to have a significant impact on many areas of our daily lives. Among other factors the steady increase in the use of AI is driven by the availability of structured large-scale data storage, often called big data (1). Big data is a key factor in AI development because machine learning algorithms take advantage of patterns present in the training data. Consequently, the size and variability of the dataset strongly impact an algorithm’s performance when deployed.

Lately, AI has become embedded in healthcare. Fields such as intensive care, radiology, and pathology gather mass amounts of data. In these areas, diagnostic support tools and disease prediction software are topics of large scientific interest (2, 3). In industry, healthcare companies are investing in AI, collaborating with hospitals and universities to accelerate research projects (4, 5). Moreover, simplification of healthcare processes with AI could potentially reduce the costs in healthcare by 5%–10% (6).

Although the impact of AI is revolutionary, its implementation in a sensitive field such as medicine requires critical evaluation and consideration. Data collection for machine learning raises moral and ethical questions: is the data collected representative of the environment in which it will be used? What will happen when an algorithm, trained on data with an over-representation of a certain group, is used on a different population? Recently, the World Health Organization (WHO) prioritized big data and artificial intelligence as one of the major topics in health ethics. In April 2020, a special issue of the WHO bulletin was released on this particular subject (7), raising concerns about responsibility, accountability, as well as lack of empathy by computers.

This manuscript focuses on a specific application of artificial intelligence (AI) in hospitals: predicting adverse events. These events, which include medication side effects, physical injury, psychological trauma, and death, represent a major concern in healthcare. Predicting such events is crucial as it allows physicians to take preemptive actions, enhancing patient safety and optimizing care delivery.

In clinical practice, substantial amounts of data are routinely collected, especially in high-risk environments such as postoperative care and intensive care units (ICUs). This data presents a valuable resource for developing predictive models. By leveraging machine learning and other AI techniques, researchers and clinicians can identify patterns and signals that precede adverse events, enabling timely interventions. Using this data, several methods have been proposed to forecast these events (8–11).

Accurate adverse event prediction has profound clinical implications. Effective models enable earlier detection of patient deterioration, prompt treatment adjustments, and optimal resource allocation, thereby reducing morbidity and mortality (8). These models also support clinical decision-making by tailoring interventions to individual patient risk profiles. However, challenges such as data quality, model interpretability, and integration into clinical workflows must be addressed (12, 13). Overcoming these obstacles can enhance predictive accuracy and reliability, allowing healthcare providers to better utilize AI for improved patient care and outcomes.

In this paper, we aim to summarize and discuss the significant challenges and key considerations involved in using AI for the prediction of critical healthcare events. Our discussion centers on issues like data biases, the lack of interpretability, the potential impact on clinical skills, and ethical questions. Given the concise nature of this mini-review, the issues and mitigation strategies discussed are selective examples from broader fields. Nevertheless, unlike other papers that address these challenges in isolation, this review offers a unified perspective, covering data biases, model interpretability, ethical concerns, and clinical integration in one accessible overview. By doing so, it serves as a practical resource for researchers and clinicians, highlighting the need for meticulous development, rigorous validation, and responsible implementation of AI in clinical decision support systems.

## 2 Search strategy

PubMed and Google Scholar were used for the research, with only articles in the English language, using terms similar to: “Clinical Decision Support,” “Artificial Intelligence” and “Adverse Events.” Articles included in this review were reviews, original papers, and opinion articles. Literature was searched from inception to February 2024. This mini-review synthesizes evidence by categorizing selected articles into thematic groups based on the application of AI in adverse event prediction, challenges faced in clinical implementation, and ethical considerations. Each article was reviewed to extract relevant challenges, solutions, and conclusions. Table 1 provides an overview of the relevant literature categorized in thematic groups.

**Table 1:** Evidence and solutions for challenges in AI implementation.

Challenges	Possible solutions
Data acquisition and preprocessing	
Bias in event defintion (14, 15)	Adapting outcome definition (15), use weighting or resampling strategies (16)
Imbalance in data (17)	Use weighting or resampling strategies (16)
Population shift (18)	Out of distribution detection (19), external validation (20, 21)
Data scarcity (22)	Resampling and/or data augmentation (16)
Missing data (23)	Remove or impute variables based on reason of missing data (23)
Bias in model selection and training	
Underfitting during model training (24)	Increase model complexity or complexity of data transforms (25, 26)
Overfitting during model training (25, 27)	Regularization (26), early stopping (28), or limiting model complexity (28)
Trust and adoption in clincal workflow	
Black box AI and lack of trust (22)	Employ explainable AI techniques (29–31)
Adoption in clinical workflow (8, 32, 33)	Integrating alerts in existing workflow (32, 33), adopting additional pathways for alerts (8)

## 3 Data acquisition and preparation

**Figure 1:**
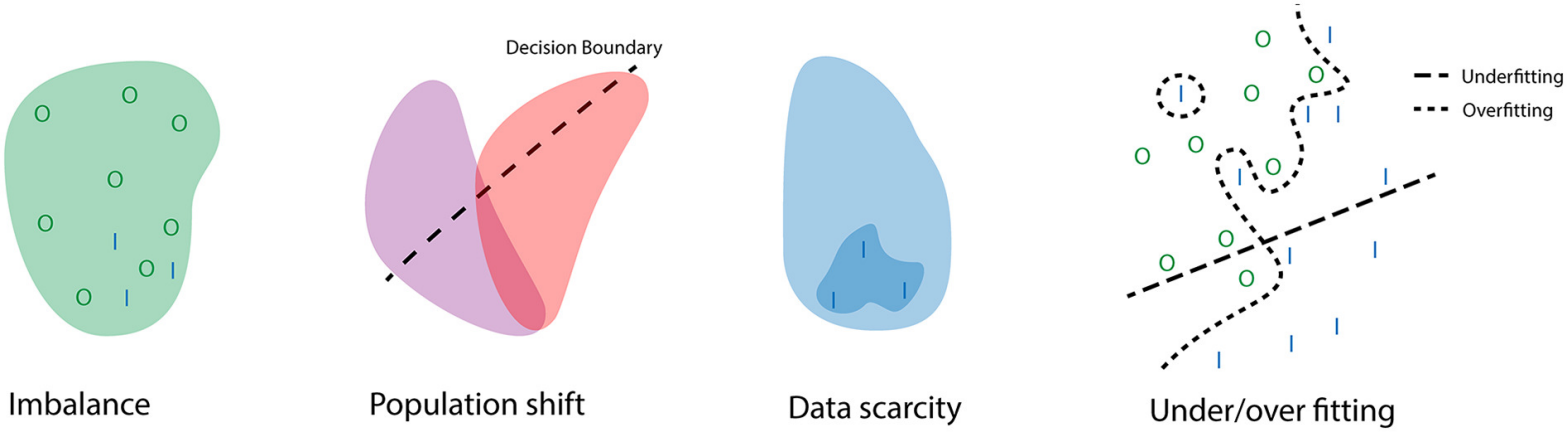
Illustration of how problems in data collection and processing can lead to errors during training. With I the positive class, and O the negative class. The example of imbalance shows how the negative class dominated the training set therefore the learned distribution reflects this even though it would still be possible for positive cases to occur. The population shift highlights how a decision boundary for one population is not always generalizable to another population. The data scarcity example illustrates how a small dataset during training can lead to wrongful assumptions of the underlying distribution. The final example displays what a decision boundary during under and overfitting might look like.

Data acquisition is critical for AI applications as learning hinges on data. Biases that are present within the data will result in biases during the learning process. In machine learning, bias can lead to erroneous assumptions created during the learning process, and illustrations of these types of errors can be found in Figure 1. These errors can arise from several factors in the learning process, the most important of which is the data. In risk prediction, biases can lead to wrongful determination of the supposed risk that a patient has of suffering an adverse event. For example, in one scenario, there is a hospital focused on cardiology that conducts numerous high-risk cardiac surgeries but only a handful of low-risk oncology surgeries. Conversely, another hospital specializes in oncology and handles many high-risk oncology surgeries. In this setup, a risk prediction model developed by the cardiology-focused hospital might show bias towards cardiac surgery, potentially leading to an underestimation of risk when applied to oncology surgeries at the second hospital.

The biggest problem in the prediction of adverse events is that these are not randomized controlled trials. Whether a patient is assigned to the control group or suffers from an adverse event is not determined randomly, but is instead a result of a multitude of factors that may or may not have been observed during the data acquisition. Additionally, the definition of adverse events is subject to change and may be dependent on local hospital practices. CDS algorithms often use mortality as the primary outcome for risk stratification (14). However, this approach may not capture the full spectrum of patient deterioration. For a more comprehensive assessment, it could be beneficial to consider other indicators of deterioration. These may include interventions such as unanticipated ICU admissions, emergency surgeries, or the administration of fluids or other medication (14, 15).

Additionally, many patients do not experience adverse events. This lack of adverse events among a large number of patients can lead to a skewed representation in the data, creating an imbalance. When a dataset is imbalanced, it means that the ratio of cases to controls is not one-to-one. If this ratio is different compared to the targeted population this is called a population or prevalence shift (17). This shift can occur because of the demographic region where the data was collected or because of the hospital population present in the data (18). The under-representation of groups in the data can in some cases be solved by weighting or resampling strategies. Applying higher weights to certain groups will cause the training process to assign higher losses to the wrong predictions of this group. Using resampling, the underrepresented group is over-sampled, and/or the over-represented group is under-sampled. This will have similar results as reweighing (16). In risk prediction models, population bias plays a large role. The best method to find and address this type of bias is through external validation. However, systematic reviews of risk prediction scores show that many of the studies regarding risk prediction do not utilize external validation (20, 21).

Population shifts pose a challenge in continuous risk prediction models. These shifts occur when a prediction model is applied to a population that does not match the underlying distribution of the training population. This might happened because of a change in hospital, hardware, laboratory protocol, drift in population overtime, etc. For instance, if a model predicting patient deterioration over time is applied in a clinical setting, resulting actions by clinicians can influence subsequent predictions. Actions deviating from the norm, prompted by risk predictions, may lead to rare occurrences not seen during training, causing inaccurate predictions. Notably, these actions aren’t necessarily incorrect, just different from most training data. It is possible to inform clinicians when this is occurring by employing out-of-distribution detection (19). This method can alert clinicians when the current data deviates from the training data, but it does not improve the model’s prediction accuracy. Prospective studies on the use of machine learning algorithms are required to increase the accuracy since these studies allow us to see if those predictions that deviate from the norm are beneficial or detrimental.

Another bias that could occur during data acquisition is data scarcity. Data scarcity means that there is too little data for the model to represent small groups or rare occurrences, leading to reduced performance and reliability. This underrepresentation is present in most studies regarding risk prediction in clinical practice since most adverse events are rare occurrences. Additionally, over 50% of the studies applying machine learning to analyze ICU data, utilized data from less than 1,000 patients (22). Such small datasets tend to overestimate performance without external validation. Remedying data scarcity ideally involves collecting more data, but that is not always feasible. Data scarcity can be mitigated by data augmentation or synthetic data generation. Data augmentation alters available data with appropriate transformations, to create a larger, diverse dataset and can also infer missing data. Synthetic data generation uses a model to generate data, potentially introducing bias depending on the underlying model assumptions.

## 4 Data preprocessing and AI training

While proper data acquisition can prevent biases in the data, improper data preprocessing and training can still result in biases. One of the most important factors is the handling of missing data. Missing data occurs mainly in three ways (23).

The first is data missing completely at random (MCAR), meaning the missing data is unrelated to any variable observed and unobserved. This is a very poor assumption, especially in risk prediction. For example, data could be missing because of faulty equipment. This process is random and therefore, not related to any variables. The second way is data missing at random (MAR), which means that the missing data is a consequence of observed variables. For example, preoperative screening data is missing, but it was observed that the patient was unconscious at hospital entry. In this case, the fact that the patient was unconscious at hospital entry could be used to infer something about the missing preoperative screening. The final is data missing not at random (MNAR), which means that the missing data is related to the unobserved variables. For example, some laboratory equipment is unable to produce a valid measurement if the value of the measurement is below a certain level. Mathematically the forms of missing data could be expressed as Equations 1–3,

(1) $$\text{MCAR:}\;P(M_{y})=P(M_{y}|X,Y)$$

(2) $$\text{MAR:}\;P(M_{y})\neq P(M_{y}|X)$$

(3) $$\text{MNAR:}\;P(M_{y})\neq P(M_{y}|Y)$$

with $M_{y}$ being the missingness of the unobserved variables, X the observed variable, and Y the unobserved variable.

Determining the cause of missing data is often impossible. Therefore, the common assumption used is missing at random, since this allows for the use of observed variables to impute the missing data. When encountering missing data, attention should be paid to the reasons why data may be missing, and if possible these reasons should be included in the data used during the training of a prediction model.

Biases can also arise during prediction model training, known as under or overfitting. Underfitting occurs when a simpler model attempts to represent a complex problem, often resulting in reduced performance in both train and test sets (27). This bias may stem from incorrect assumptions about the data. For instance, logistic regression models assume linear relations between input and output variables, leading to underfitting in complex problems with non-linear relations between variables (25). Chances of underfitting can be reduced by utilizing data transforms or machine learning models which can represent and fit data distributions of higher complexity (25).

Excessive use of complex models may lead to overfitting, the opposite of underfitting. In overfitting, the learning process identifies relationships in the training data that don’t generalize to the test set, resulting in performance disparities (24). Specifically, the model may perform better on the training set than on the test set. Overfitting can be reduced through regularization. Regularization imposes constraints or penalties during training on the complexity of the method, such as adding cost terms to model weights. This encourages the model to increase only the weights of important features (26). Early stopping is another regularization method where the training set is split into a training-validation split, and training is halted when the validation loss begins to increase (28). Additional regularization methods for tree-based models often involve limiting the depth of the trees (28).

Besides complexity, another and perhaps more important aspect of model selection is the intended application. As discussed previously, CDS algorithms focus on the prediction of adverse events. This can be a prediction made at a specific moment, but more often it is used to create predictions along longitudinal data (15, 34). Cascarano et al. provide a comprehensive review of various methods for applying AI to longitudinal biomedical data (34). The appropriateness of these methodologies is determined based on the nature of the input and output data. This approach allows for a more targeted and effective application of AI in CDS.

## 5 Interpretability and trust

AI can suffer from a lack of interpretability. This means it is not easy to determine why a model would for example assign a higher risk to one patient over another. The current clinical practice employs scoring cards such as the early warning score (EWS), which assigns a single score to a few chosen variables which together add up to a warning score (35). For these types of methods, determining the relevance of each variable is rather straightforward. However, when using more complicated methods, the influence of a single variable could be dependent on one or multiple other variables. This becomes even more complex when employing models containing hidden parameters such as hidden Markov models or (deep) neural networks. These parameters indirectly influence the input or output variables, instead of individual variables. Although these methods are becoming more commonly used because they can achieve higher performance when dealing with complex problems they inherently suffer from a lack of interpretability (22).

The primary problems created by the black-box nature of machine learning are mistrust and the lack of insight in the cases the model breaks down. A clinician will need to trust blindly that the algorithm will give accurate results since reasoning how the algorithm came to a specific conclusion is difficult. There are interpretation methods that aim at achieving explainable AI (29, 30). In general, these methods are subdivided into global or local interpretations. Global methods attempt to explain the general behavior of the model by observing distributions or determining the influence of features on the overall performance. Local methods attempt to interpret how features affect individual predictions. However, these algorithms do not answer the question of why the relevant features influence the outcome. That is where clinical interpretation remains key. For (deep) neural networks, other interpretation methods are required since the input data is often raw data instead of engineered features. These interpretation methods work by determining the features that the network encodes in the latent space, finding the relevant raw data (attention maps), or making small changes in the raw data until the model breaks (31). These methods are especially useful for highlighting important artifacts in the raw data. Nevertheless, algorithms utilizing machine learning will still need to be tested rigorously to eliminate any form of bias.

The best practice would be for every hospital to test an algorithm on their specific hospital population before deciding to implement it in clinical practice. The resulting performance will be an indication of how reliable this algorithm could be in a real-world pilot. However, performance should not be the only metric to judge risk prediction algorithms. A more important metric for risk prediction algorithms or, more in general, for clinical decision support systems is the clinical outcome. Evaluating clinical outcomes is, however, more challenging, as it requires the design of a prospective study that employs a randomized control trial to assign patients in a case group, for which the algorithm is used for the clinical decision-making, and a control group for which standard clinical practice is used. If a significant improvement in the clinical outcome can be shown by using AI, the acceptance of AI algorithm could greatly improve.

## 6 Clinical translation

Successful implementation of validated medical prediction algorithms into healthcare environments requires more than just technical functionality. Beyond the algorithms’ accuracy and performance, these implementations involve an array of considerations, spanning ethical, clinical, societal, and organizational facets.

From a clinical standpoint, the adoption of AI tools involves thoughtful integration into existing healthcare workflows. Health professionals must be trained not only in the use of such tools, but also in the interpretation of the algorithm’s outputs, considering the potential for nuanced clinical situations that may require human intervention. Such implementation may necessitate the development of new clinical protocols, in which AI tools are integrated into decision-making processes, while still retaining a space for the clinician’s judgment. Studies by Escobar et al., Kollef et al., and Evans et al. show how automated early warning scores can be integrated into rapid response team (RRT) alerts (8, 32, 33). The authors employed a nurse who would transfer actionable data to RRTs based on alerts by an automated early warning score. These kinds of integrations ensure that the AI is leveraged as an aid, not as a replacement for human expertise. Moreover, seamless integration should account for data security, privacy, and interoperability with existing systems, given the highly sensitive nature of healthcare data. These aspects are key to building trust with clinicians and patients alike and to compliance with various data protection regulations. Additionally, it is important that these algorithms are not a burden on the medical staff, for example by further increasing alarm fatigue. In the abovementioned studies, this was achieved by setting the thresholds of alarms at a rate that was deemed acceptable for the nursing staff.

Another important aspect of clinical translation is trust in the algorithms. Often transparency of the algorithm is brought up as a way to increase trust in AI algorithms. However, transparency in complex algorithms often comes at the cost of simplification, which could hurt performance and thereby create a false form of trust. Instead, we should aim at rigorous testing of AI algorithms, before implementation. Similar to how drugs with unknown mechanisms of action are still used in clinical practice after thorough investigation (36).

The adoption of AI tools in healthcare may also raise questions about the potential deskilling of healthcare practitioners (37). If AI algorithms are increasingly used to carry out diagnostic or prognostic tasks, it is possible that clinicians’ skills in these areas might diminish over time. There is also the risk that over-reliance on AI tools may lead to complacency, causing clinicians to overlook or misinterpret signs that the AI might miss. It therefore seems important that training for healthcare professionals emphasizes the continued importance of their own judgment and clinical skills.

Finally, evaluation processes must be put in place to assess the real-world effectiveness and utility of AI tools post-implementation. This involves regular review and iteration of the algorithms, addressing any discovered biases or inconsistencies, and assessing user satisfaction and overall system impact. These reviews will ensure that AI prediction tools remain clinically relevant, ethically sound, and beneficial to both patients and healthcare practitioners.

AI regulation in healthcare is an evolving landscape, largely focusing on privacy, data protection, and the safety of AI systems. Regulatory frameworks like the Health Insurance Portability and Accountability Act in the U.S. and the General Data Protection Regulation and AI Act in the E.U. provide guidelines for legally compliant deployment of AI. However, as these regulations were not initially designed for AI in medicine, their adequacy for AI-driven healthcare solutions is subject to ongoing discussions (38). Moreover, the increasing prevalence of AI in healthcare prompts considerations around accountability for AI errors, transparency of its operations, potential algorithmic biases, and implications for patient consent and autonomy. Policymakers worldwide should address these unique challenges of AI through adjustments to existing laws or the formulation of new ones, aiming for a balanced approach that promotes innovation, builds trust, safeguards patient rights, and ensures the safe and effective use of AI in healthcare.

## 7 Limitations

This review has several limitations. Its brevity restricts in-depth exploration of technical or ethical nuances, and it does not extensively address unique challenges of specific AI approaches, such as large language models (LLMs) or deep learning. Due to the limited scope, the discussion is not exhaustive but aims to highlight significant areas for further exploration. Additionally, while ethical and regulatory considerations are highlighted, practical guidance on navigating these issues is limited.

## 8 Conclusion

The implementation of AI for predicting adverse events in healthcare is a complex endeavor that demands meticulous attention to data quality, preprocessing, model training, interpretability, and ethical considerations. Clarity on the populations used in the development of AI-driven tools can expose certain biases and proper use of regularization techniques can improve the generalizability of CDS algorithms outside their original population. Rigorous testing of these tools before implementation is required to build trust. In this regard, black-box AI algorithms can be treated the same as drugs with unknown mechanisms of action, as both require randomized controlled trials to ensure that their use achieves the desired effect. Implementation will also require the training of healthcare professionals not only to ensure the effective use of the algorithms but also to prevent deskilling. Addressing these challenges is essential to realizing the transformative potential of AI while ensuring its responsible and trustworthy integration into clinical decision-making processes.
